# Variations of Major Glucosinolates in Diverse Chinese Cabbage (*Brassica rapa* ssp. *pekinensis)* Germplasm as Analyzed by UPLC-ESI-MS/MS

**DOI:** 10.3390/ijms25094829

**Published:** 2024-04-29

**Authors:** Seong-Hoon Kim, Kingsley Ochar, Kanivalan Iwar, Yoon-Jung Lee, Hae Ju Kang, Young-Wang Na

**Affiliations:** 1 National Institute of Agrobiodiversity Center, National Institute of Agricultural Sciences, Rural Development Administration, Jeonju 5487, Republic of Korea; ocharking@korea.kr (K.O.); kani05@korea.kr (K.I.); yoon112@korea.kr (Y.-J.L.); ywna@korea.kr (Y.-W.N.); 2 Department of Agrofood Resource, National Institute of Agricultural Sciences, Rural Development Administration, Jeonju 5487, Republic of Korea

**Keywords:** Chinese cabbage, Germplasm, Glucosinolate, variability, Diversity, HPLC

## Abstract

In this study, the variability of major glucosinolates in the leaf lamina of 134 Chinese cabbage accessions was investigated using Acquity ultra-performance liquid chromatography (UPLC-ESI-MS/MS). A total of twenty glucosinolates were profiled, of which glucobrassicanapin and gluconapin were identified as the predominant glucosinolates within the germplasm. These two glucosinolates had mean concentration levels above 1000.00 μmol/kg DW. Based on the principal component analysis, accessions IT186728, IT120044, IT221789, IT100417, IT278620, IT221754, and IT344740 were separated from the rest in the score plot. These accessions exhibited a higher content of total glucosinolates. Based on the VIP values, 13 compounds were identified as the most influential and responsible for variation in the germplasm. Sinigrin (*r* = 0.73), gluconapin (*r* = 0.78), glucobrassicanapin (*r* = 0.70), epiprogoitrin (*r* = 0.73), progoitrin (*r* = 0.74), and gluconasturtiin (*r* = 0.67) all exhibited a strong positive correlation with total glucosinolate at *p* < 0.001. This indicates that each of these compounds had a significant influence on the overall glucosinolate content of the various accessions. This study contributes valuable insights into the metabolic diversity of glucosinolates in Chinese cabbage, providing potential for breeding varieties tailored to consumer preferences and nutritional demands.

## 1. Introduction

Phytochemicals, the bioactive compounds in plants exhibit a remarkable biochemical repertoire that contributes to plant adaptation, defense mechanisms, and interactions with their environment [1]. The abundance and complexity of these metabolites is also indicative of the enormous potential for discovering novel bioactive compounds and their applications in various fields such as medicine, agriculture, and industry [2]. Many profiled phytochemicals, including glucosinolates, play key roles in supporting human health [3]. Glucosinolates (GSLs), known as β-thioglucoside-N-hydroxysulfates or S-glucopyranosyl thiohydroximates, are sulfur-containing compounds mainly abundant in *Brassica* species [4,5]. The chemistry of GSLs encompasses a core structure composed of a β-D-thioglucose group, a sulfonated oxime group, and a side chain derived from amino acids [6,7]. Based on their amino acid precursors and distinct biosynthesis process, GSLs may be considered as aliphatic, aromatic, and indole compounds [8,9]. Aliphatic glucosinolates originate from methionine, alanine, leucine, isoleucine, or valine; aromatic glucosinolates are derived from phenylalanine or tyrosine; and indole glucosinolates originate from tryptophan. When *Brassica* tissue is damaged, through processes including chewing and crushing, myrosinase catalyzes the enzymatic hydrolysis of GSLs and produces bioactive breakdown products such as isothiocyanates (ITCs), nitriles, epithionitriles, or thiocyanates. [10]. The diversity of GSLs is the main factor contributing to variability or the distinct flavors and nutritional composition of *Brassica* plants [11]. Evidence from previous research indicates that certain GSLs have a great potential as health-promoting agents, particularly their role as anti-inflammatory, anti-oxidation, and cancer chemo-protective molecules [10,12].

*Brassica rapa* (AA; 2n = 20), a diploid *Brassica* species exhibits, enormous morphological diversity stemming from both natural variations and human-driven selections, thus revealing a wide spectrum of characteristics across different subspecies including Chinese cabbage [13,14]. Chinese cabbage (*Brassica rapa* L. ssp. *pekinensis*) is a green leafy vegetable mainly consumed in Asian and some Western countries. This vegetable forms the main ingredient of kimchi, a salted and fermented traditional dish in the Republic of Korea [15,16]. As a result of the diverse essential metabolites inherent in the crop, it has gained significant attention in diverse fields of study including agricultural, medical, and nutritional sciences on account of its rich health-promoting GSL content [17]. The presence of diversity in *Brassica* genetic resources allows for the exploration and identification of a broad spectrum of targeted metabolites, and identifying the extensive biochemical variability within different plant species and accessions. The GSLs profiles differ significantly among different accessions of Chinese cabbage due to factors such as genetic polymorphisms, environment, developmental stage and geographical origins [10,18,19]. Unraveling the diversity of GSLs content across diverse germplasm collections is crucial to pave the way for elucidating the metabolic pathways governing their biosynthesis and also for harnessing this variation in breeding programs that target developing superior cultivars with augmented nutritional quality and health-promoting attributes [8,20]. Such diversity also enables researchers to uncover and analyze a wide array of bioactive compounds, facilitating a deeper understanding of GSLs and their potential applications in various fields, including agriculture, nutrition, and medicine. Therefore, this study aimed to determine variations in the major glucosinolates in diverse Chinese accessions using an Acquity UPLC-ESI-MS/MS system.

The current study investigated variations in major glucosinolates (GSLs) in a wide range of Chinese cabbage (*Brassica rapa* ssp. *pekinensis*) germplasm using UPLC-ESI-MS/MS, a method offering precise profiling. By analyzing a larger number of accessions compared to previous studies, the research provides a comprehensive understanding of GSL diversity in Chinese cabbage. These findings provide useful information for designing breeding strategies to develop cultivars with optimized GSL content, enhancing their nutritional quality and potential health benefits for human consumption.

## 2. Results and Discussion

### 2.1. Identification and Quantification of Glucosinolates in Chinese Cabbage

In this study, the multiple reaction monitoring (MRM) mode was used for the detection of all 20 of the GSLs. This involved monitoring specific transitions that generated characteristic fragment ions, providing detailed and specific information about the GSLs present. The twenty distinct GSLs were profiled, consisting of thirteen aliphatic (65%), four aromatics (20%), and three indoles (15%) GSLs. Details of the various GSLs, including their semi-systematic names, molecular weight, and class are given in Table 1. These results are in agreement with previous reports that aliphatic GSL are generally the most abundant GSLs in *Brassica* vegetables [21]. Earlier Lee et al. [20] noted that 68% of the total GSLs identified in 62 varieties was in aliphatic class, including five aliphatic, one aromatic, and four indole compounds.

Table 2 highlights the significant variability in glucosinolates within the 134 accessions. Two aliphatic GSLs, glucobrassicanapin and gluconapin, were dominant among all the individual GSLs identified, with a mean GSL content greater than 1000.00 μmol/kg DW compared to the rest of the compounds. This was followed by progoitrin, epigoitrin, and glucoalyss in. This is in agreement with previous findings indicating that glucobrassicanapin and gluconapin are the GSLs that are most frequently detected in the *Brassica* germplasm [20,22,23]. Gluconasturtiin and 4-Methoxyglucobrassicin were the predominant GSLs among the aromatic and indole groups, respectively. In their study, Kim et al. [5] investigated the composition and content of GSLs in 24 varieties of Korean *Brassica rapa* L. ssp. *pekinensis* and found glucobassicanapin, 4-methoxyglucobrassicin, gluconapin, and glucobrassicin as the predominant GSLs detected. In another study, Chen et al. [24] compared the GSL composition in five species of the Chinese flowering cabbage *Brassica campestris* and noted that glucobrassicin was the major GSL in the crop. Chun et al. [25] also explored glucosinolates and carotenoids contents in eleven Chinese cabbage varieties and identified 13 GSLs, encompassing progoitrin, sinigrin, glucoalyssin, gluconapoleiferin, gluconapin, glucocheirolin, glucobrassicanapin, glucoerucin, 4-hydroxyglucobrassicin, glucobrassicin, 4-methoxyglucobrassicin, neoglucobrassicin and gluconasturtiin which were also detected in our present list of GSLs. They noted that the contents of gluconapin and glucobrassicanapin (54% and 22%, respectively) were relatively higher in the 11 Chinese cabbage varieties, which agrees with earlier reports that aliphatic compounds, with four-carbon (4C) and five-carbon (5C) side chains of methionine, are the most abundant GSLs in *Brassica rapa* such as Chinese cabbage [26,27]. Also, in a quantitative analysis of the GSLs in the seeds and edible parts of Korean Chinese cabbage, the concentrations of 4-Methoxyglucobrassin and progoitrin predominated the individual GSLs identified [28]. In concordance with this finding, 4-Methoxyglucobrassicin was among the most highly expressed GSLs detected in the current study. The observed variability in GSL content within the germplasm underscores the substantial influence of both genetic and environmental factors on the biosynthesis and expression of GSLs in *Brassica* species as highlighted in a previous study [29]. Generally, the total GSL content varied significantly among the accessions, ranging from 626.53 in the cultivar IT344764 to 21,199.02 μmol/kg DW in the landrace IT120044 (Supplementary Materials Table S1). Besides these accessions, IT186728, IT221789, IT221754, IT278620, and IT100417, with a total GSL content of 18009.37 μmol/kg DW, 16,680.10 μmol/kg DW, 15,549.54 μmol/kg DW, 14,007.51 μmol/kg DW, and 13,869.67 μmol/kg DW, respectively, were comparatively higher. Accessions IT221733 (689.61μmol/kg DW), IT214688 (917.50 μmol/kg DW), IT278703 (1259.31 μmol/kg DW), and IT336315 (1432.55 μmol/kg DW) exhibited the lowest concentrations of total GSLs (Supplementary Materials Table S1). In this study, the GSL profile varied substantially from previously reported concentration levels [5,20,25,30]. This disparity in GSLs composition may be due to differences in plant materials used, implying that evaluating a wide diversity of germplasm is highly requisite. Overall, these findings are useful for future development of *Brassica* varieties with tailored glucosinolate profiles aligned with consumer preferences.

### 2.2. Multivariate Analysis

**Figure 1:**
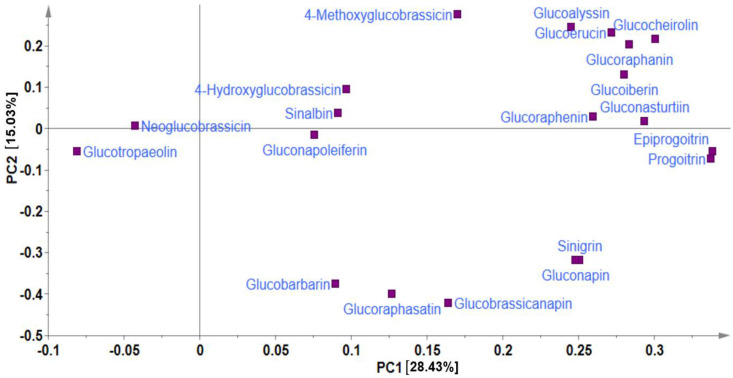
Principal component analysis showing loading plot of PC1 against PC2.

**Figure 2:**
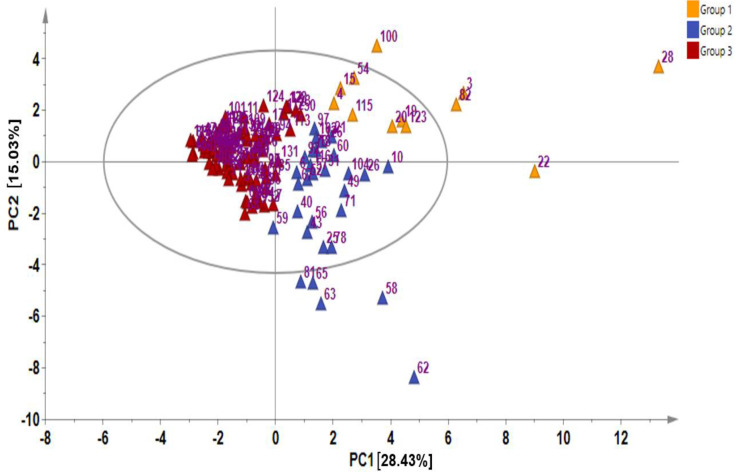
Principal component analysis showing score plot of the 134 Chinese cabbage accessions.

In metabolite analyses, particularly those involving crop genetic resources, the combination of PCA and HCA approaches provides a useful means of elucidating the chemical relationships and variations within diverse germplasms, providing valuable insights into their identification and chemo-classification based on their metabolite compositions [21,31]. Using the PCA, the main sources of variability in the GSL content across all the 134 Chinese cabbage accessions were visualized, identifying the respective contribution of each compound to the variability of the germplasm. The two highest-ranking PCs, PC1 (28.43%) and PC2 (15.03%) collectively accounted for 43.46% of the total variance (Figure 1 and Figure 2).

In PCA, factor loading represents the connection between the variables and principal components, indicating both the strength and direction of their relationships. The factor loading comparison in PC1 was used to identify the GSLs that mainly contributed to the total variation. Comparing the metabolite loadings in PC1 and PC2 to understand the main GSLs contributing to variability in the Chinese cabbage germplasm, the results revealed that almost all of the GSLs (except neoglucobrassicin and glucotropaeolin) showed a higher weight in PC1, explaining 28.43%of the total variance, the largest proportion (Figure 1 and Figure 2). The PCA separated all the accessions into three distinct groups based on their GSL content as indicated by yellow, blue, and red triangles (Figure 2). Group 1 contained accessions including IT186728, IT120044, IT278620, IT100417, IT344740, and IT344768 which highly corrected with glucocheirolin, gluconasturtiin, glucoraphanin, glucoiberin, glucoerucin, glucoalyssin, and glucoraphenin. Group 2 consisted of several accessions, including IT221789, IT221754, IT227012, IT260815, and IT227906, with higher contents of compounds including sinigrin, gluconapin, glucobrasicanapin, glucoraphasatin, and glucobarbarin. Group 3 comprised the majority of the accessions (Figure 2). This group showed a characteristically higher content of neoglucobrassicin and glucotropaeolin. Generally, the results revealed the presence of distinct variation among the germplasms based on their GSL content, providing an excellent opportunity for future breeding for improved GSL content in Chinese cabbage. Accessions, including IT186728, IT120044, IT221789, IT100417, IT278620, IT221754, and IT34474,0 had higher contents of total GSL and several individual GSLs and were distinctly separated from the rest (Figure 2). Generally, the PCA did not reveal any clear separations between the accessions based on accession type and origin, implying that the genetic diversity observed among the accessions was not strictly defined by these factors. A comparable pattern was also reported in another study where the metabolic profiles of 38 Chinese cabbage cultivars were investigated for their GSL and carotenoid composition [30].In addition, the clustering of the accessions was not influenced by the accession type, cultivar, or landrace and origin. Thus, other genetic and environmental factors may play a more significant role in shaping the observed patterns of diversity, underscoring the complexity of the genetic relationships within the studied accessions.

**Figure 3:**
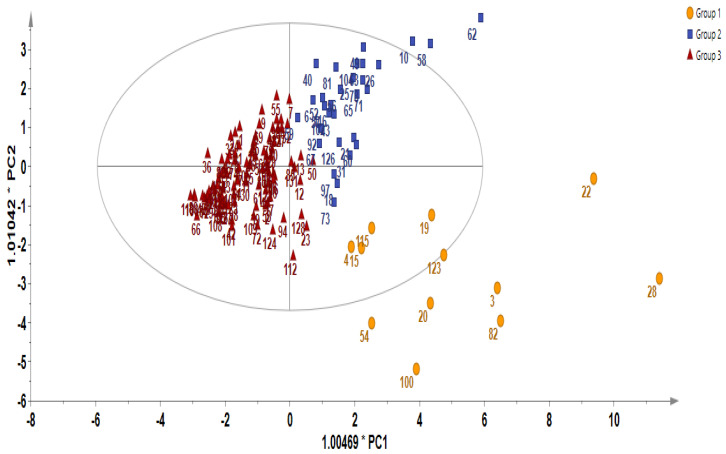
Diversity analysis showing Orthogonal Partial Least Squares Discriminant Analysis (OPLS-DA).

**Figure 4:**
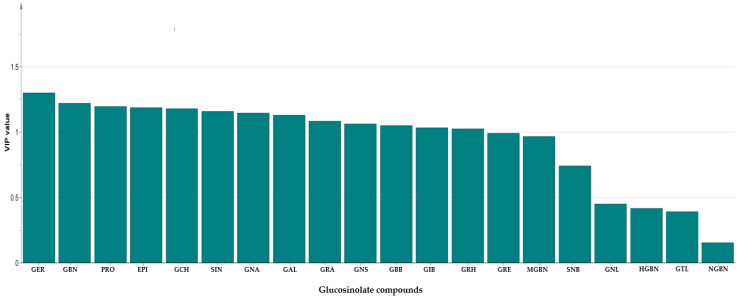
Variable Importance in Projection (VIP) value showing most influential glucosinolates in the 134 Chinese cabbage accessions. GER: Glucoerucin; GBN: Glucobrassicanapin; PRO: Progoitrin; EPI: Epiprogoitrin; GCH: Glucocheirolin; SIN: Sinigrin; GNA: Gluconapin; GAL: Glucoalyssin; GRA: Glucoraphanin; GNS: Gluconasturtiin; GBB: Glucobarbarin; GIB: Glucoiberin; GRH: Glucoraphasatin; GRE: Glucoraphenin; MGBS: 4-Methoxyglucobrassicin; SNB: Sinalbin; GNL: Gluconapoleiferin; HGBN: 4-Hydroxyglucobrassicin; GTL: Glucotropaeolin; NGBS: Neoglucobrassicin.

In order to gain additional insight into the diversity present in the germplasm, the supervised Orthogonal Partial Least Squares Discriminant Analysis (OPLS-DA) was used to identify the key accessions that contributed significantly to the cluster differentiation. Based on the OPLS-DA results, three separate groups of accessions were obtained as indicated in Figure 3 as yellow circles, blue rectangles, and red triangles. Group 1 consisted of the least number of accessions (a total of 12), encompassing IT186728, IT120044, IT278620, IT100417, IT344740, IT120042, IT221742, IT344768, IT120040 IT344759, IT120022, and IT100422. Accessions including IT221789, IT221754, ITIT112664, IT166986), IT215001, IT260815, IT247932, IT120036, and IT344735 constituted Group 2. Group 3 comprised the majority of the accessions. These results reveal the presence of distinct variation within the germplasm based on their GSL content. Figure 4 indicates individual GSLs and their contributions to the three clusters based on their VIP values. Variables with VIP values greater than one are the most significant contributors to the observed variability [8,32]. Based on the VIP values, 13 out of the 20 GSLs were identified as the most influential compounds responsible for variation in the Chinese cabbage germplasm. These compounds were glucoerucin, glucobrassicanapin, progoitrin, epiprogoitrin, glucocheirolin, sinigrin, gluconapin, glucoalyssin, glucoraphanin, gluconasturtiin, glucobarbarin, glucoiberin, and glucoraphasatin.

Further, in order to verify the segregation pattern of the GSLs within the accessions, the metabolites data weresubjected to heatmap hierarchical clustering analysis (Supplementary Materials Figure S1). The heatmap revealed three different groups of accessions, with varied patterns of GSL concentration being observed. In line with the results of the PCA, some accessions, including IT186728, IT120044, IT2786208, IT100417, IT221742, IT344740, IT344759, and IT120040, were clustered together. Specifically, accession 28 exhibited relatively higher content of glucoraphanin, glucocheirolin, glucocoiberin, epigrogoitrin, and progoitrin while accessions 82 showed higher expression of epiprogoitrin, progoitrin, glucoerucin, gluconasturtiin, and glucoalyssin. Accession 22 exhibited a high content of several GSLs such as glucoiberin, glucoraphenin, epiprogoitrin, progoitrin, and gluconasturtiin. Similarly, accessions such as IT221789, IT221754, IT112664, IT227012, IT227906, and IT235966 were grouped together in the second group, while the third group consisted of a larger number of accessions including IT214688, IT228181, IT344756, IT344757, and IT344777. The concentrations of glucobrassicanapin and gluconapin were detected in higher amounts across the majority of the accessions. Other GSLs which individually showed a high GSL content in the germplasm included glucoalyssin, 4-Methoxyglucobrassicin, epigoitrin, and progoitrin.The results of the heatmap alsoclearly revealed theGSLs which were less expressed across the germplasm, including sinalbin, glucoiberin, glucoraphasatin, glucoraphenin, glucotropaeolin, sinigrin, and glucocheirolin.

### 2.3. Correlation Analysis of Glucosinolates

**Figure 5:**
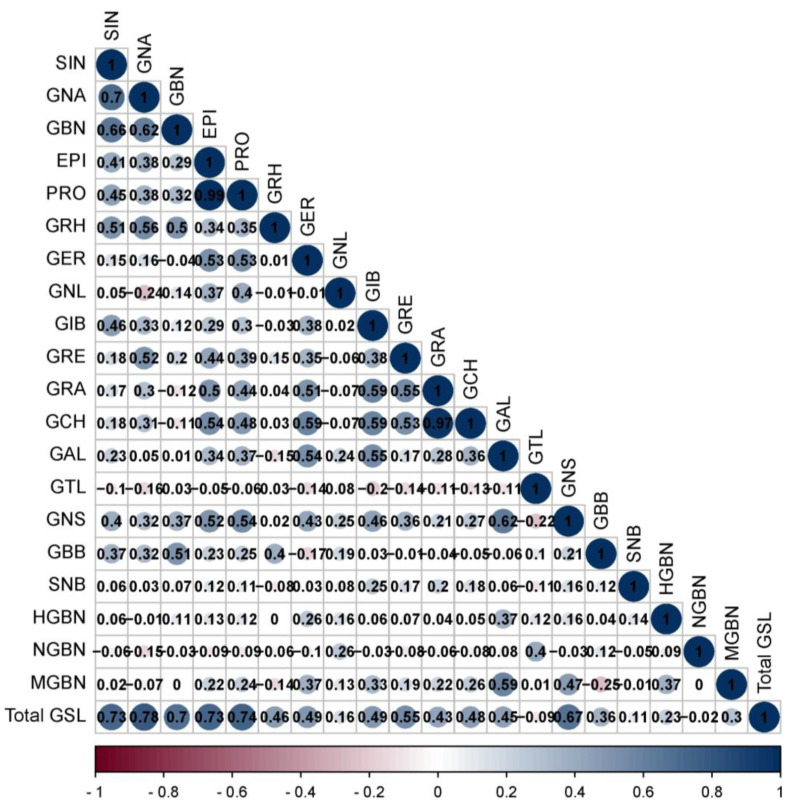
Pearson’s correlation coefficient among individual and total glucosinolate. The bigger the circle the stronger and higher the significance. SIN: Sinigrin; GNA: Gluconapin; GBN: Glucobrassicanapin; EPI: Epiprogoitrin; PRO: Progoitrin; GRH: Glucoraphasatin; GER: Glucoerucin; GNL: Gluconapoleiferin; GIB: Glucoiberin; GRE: Glucoraphenin; GRA: Glucoraphanin; GCH: Glucocheirolin; GAL: Glucoalyssin; GTL: Glucotropaeolin; GNS: Gluconasturtiin; GBB: Glucobarbarin; SNB: Sinalbin; HGBN: 4-Hydroxyglucobrassicin; NGBS: Neoglucobrassicin; MGBS: 4-Methoxyglucobrassicin; TGSL: Total GSL.

In addition to the PCA, the correlation coefficient (*r*) provides valuable insights into the complex metabolic network governing the biosynthesis of compounds and helps to reveal existing associations between metabolic signals [8,33,34]. Understanding the serelationships is crucial for a targeted manipulation that aims to alter the level of production of metabolic compounds in breeding programs. Two GSLs being positively correlated suggest a likelihood of shared biological pathway, and if the content of one GSL is enhanced, the level of expression of the corresponding compound might inadvertently increase [8]. In this study, the correlation analysis highlighted significant biochemical relationships among various GSLs in the Chinese cabbage, with both positive and negative correlations being observed (Figure 5).

In particular, certain aliphatic and indole glucosinolates exhibited strong positive correlations, indicating their potential co-regulation or shared biosynthetic pathways. The compounds sinigrin (*r* = 0.73), gluconapin (*r* = 0.78), glucobrassicanapin (*r* = 0.70), epiprogoitrin (*r* = 0.73), progoitrin (*r* = 0.74), and gluconasturtiin (*r* = 0.67) showed strong positive correlations with the total GSLs at *p* < 0.01 (Figure 5), emphasizing the substantial influence of these compounds on the overall glucosinolate content in Chinese cabbage. Apart from gluconasturtiin, which is an aromatic GSL, the remaining compounds belong to the aliphatic class, implying that aliphatic GSLs are the main indicators of total GSLs in Brassicas. Similarly, glucoraphasatin (*r* = 0.46), glucoerucin (*r* = 0.49), glucoiberin (*r* = 0.49), glucoraphenin (*r* = 0.55), glucoraphanin (*r* = 0.43), glucocheirolin (*r* = 0.48), and glucoalyssin (*r* = 0.45) exhibited significantly positive associations with the total GSLs at *p* < 0.01. Compared with other compounds, the strongest correlation was observed between progoitrin and epiprogoitrin (*r* = 0.99, *p* < 0.01), and glucocheirolin and glucoraphanin (*r* = 0.97, *p* < 0.01). The results also revealed a significantly (*p* < 0.01) stronger positive correlation between progoitrin and epiprogoitrin (*r* = 0.99), glucocheirolin and glucoraphanin (*r* = 0.97), gluconapin and sinigrin (*r* = 0.7), glucobrassicanapin and sinigrin (*r* = 0.66), glucobrassicanapin and gluconapin (*r* = 0.62), and gluconasturtiin and glucoalyssin (*r* = 0.619). A strong association (*p* < 0.01) among GSLs belonging to the same class was observed, such as aliphatic compounds (progoitrin and epiprogoitrin (*r* = 0.99), glucocheirolin and glucoraphanin (*r* = 0.97), gluconapin and sinigrin (*r* = 0.70), and glucobrassicanapin and sinigrin (*r* = 0.66). The current findings elucidated the intricate interplay between glucosinolates and provided valuable insights into the potential co-regulation mechanisms, offering a foundation for targeted breeding strategies to modulate glucosinolate profiles in the crop.

## 3. Materials and Methods

### 3.1. Chemicals and Reagents

Analytical-grade chemicals and solvents were purchased from Fisher Scientific Korea Ltd. (Seoul, Republic of Korea) and Sigma-Aldrich (St. Louis, MO, USA) for the analyses. The 20 GSL standards including gluconapin, glucobrassicanapin, progoitrin, epiprogoitrin, gluconapoleiferin, gluconasturtiin, glucoalyssin, glucoraphanin, glucoerucin, glucocheirolin, sinigrin, glucotropaeolin, glucoraphenin, glucoraphasatin, glucoiberin, sinalbin, glucobarbarin, 4-Hydroxyglucobrassicin, neoglucobrassicin, and 4-Methoxyglucobrassicin) with a purity level ≥ 97% were procured from PhytoplanDiehm & Neuberger GmbH (Heidelberg, Germany).

### 3.2. Plant Materials, Experimental Condition and Sampling

**Figure 6:**
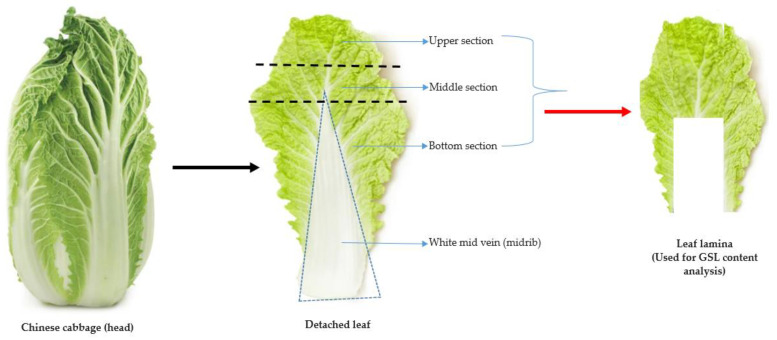
The procedure used for sampling the leaf lamina (red arrow) for the GSL content analysis of Chinese cabbage germplasm.

In this study, a total of 134 Chinese cabbage accessions obtained from the Rural Development Administration (RDA) Genebank located at the National Agrobiodiversity Center (Jeonju, Republic of Korea) were used as the materials for the GSL analyses. The accessions originated from 23 different geographical locations, offering a broad representation of the genetic diversity inherent in *Brassica* species. Detailed information on the various accessions, including individual IT numbers, accession name, accession type, and origin is indicated in Supplementary Materials Tables S1 and S2. The cultivation and experimental procedures were performed at the research farm located in the National Agrobiodiversity Center, Jeonju, Republic of Korea (35°49′18′′ N 127°08′56′′ E). Plant cultivation method was similar to previously described methodology [35], where seedlings of each of the accessions were grown under greenhouse conditions. At an optimal growth stage, four weeks after seed sowing, seedlings displaying uniformity and vigor and4 to 6 well-developed leaves were transplanted into an experimental field. Each accession, consisting of 25 individual plants, was transplanted onto beds measuring 60 × 40 cm. Adherence to appropriate agricultural practices [35] encompassing nutrient supplementation, optimal irrigation, and essential cultural practices were observed throughout the cultivation process to raise vigorous and healthy plants for glucosinolate (GSLs) content analyses. Plant harvest was carried out in the second week of November 2022. For each of the 134 Chinese cabbage accessions, the leaf lamina (and leaf blade), encompassing the upper, middle, and bottom parts of the leaf, was separated from the white midvein (or midrib) [36,37]. The leaf lamina parts were then used as material samples for the GSL content analysis (Figure 6). To preserve the integrity and safeguard the stability of the GSLs of each sample and avoid any potential enzymatic degradation of the GSLs, an immediate freezing process at subzero temperatures was carried outpost-harvest until further processing.

### 3.3. Sample Pretreatment and Extraction

The harvested samples were stored in vinyl freezer bags and immediately preserved at −80 °C to maintain their integrity until processing. To remove the moisture content, the frozen samples were lyophilized for 48 h using a LP500 vacuum freeze drier (Ilshinbiobase Co., Seoul, Republic of Korea). The lyophilized samples were then ground to a fine powder-like consistency, and preserved at −80 °C until ready for further analysis. GSLs extraction was performed similar to the protocol as described by Rhee et al. [36]. In brief, 0.1 g of the sample was mixed with 5 mL of 80% methanol and allowed to incubate at 25 °C for 30 min, followed by agitation at 120 rpm for an additional 30 min at room temperature. Subsequently, the mixture was centrifuged with a VS-180CFi centrifuge (Vision Scientific Co., Daejeon, Republic of Korea) at 14,000 rpm, 4 °C, for 10 min. To ensure a precise quantification and characterization of the glucosinolate content in the samples, the resulting supernatant, containing the extracted GSL was promptly transferred to a vial for immediate analysis using UPLC-MS/MS.

### 3.4. The UPLC-MS/MS Analysis Condition and Identification of GSLs

The UPLC-MS/MS analysis of GSLs was carried out using Waters ACQUITY^TM^ ultra performance liquid chromatography system (Waters Corp., Milford, CT, USA), coupled to the Xevo™ TQ-S Mass system (Waters MS Technologies, Manchester, UK). Chromatographic separation was performed during the analysis, using the ACQUITY UPLC BEH C18 column (1.7 μm, 2.1 × 100 mm, Waters Corp., Manchester, UK). The analysis was conducted at a constant flow rate of 0.5 mL/min, utilizing a column temperature maintained 35 °C, and an injection volume of 5 μL. In the mobile phase, phase A consisted of 0.1% aqueous solution of trifluoroacetic acid; phase B consisted of 0.1% trifluoroacetic acid in methanol. The elution conditions involved initial condition fixed at 100% of A; 0.0–1.0 min, 100% of A; 1.0–7.0 min, 100 to 80% A; 7.0–10 min, 80 to 0% of A; 10–11 min, 0 to 100% of A; 11–15 min, 100% of A. The mass spectrometry instrument functioned in negative ion electrospray ionization (ESI^−^) mode, utilizing multiple reactions monitoring (MRM). The MassLynx 4.1 software facilitated data acquisition throughout the experiment. The MS/MS detection involved specific ionization source parameters: capillary and cone voltages were set at 3kV and 54 V, and the ion source and desolvation temperatures were set at 150 and 350 °C, respectively. Additionally, the cone and desolvation gas flowed at rates of 150 and 650 Lh^−1^, ensuring optimal conditions for ionization and desolvation processes during analysis. Identification of glucosinolates (GSLs) was established by comparing their retention times and MS/MS fragmentation spectra with reference to the commercially available standards, ensuring accurate compound identification. The MRM, utilizing a specific MS/MS transition for each compound was used to identify individual GSLs. The MRM parameters were selected to optimize the detection and identification of the targeted GSLs. Estimation of final GSL concentrations involved linear regression equations derived from calibration curves generated with corresponding standards, thus permitting accurate quantification.

### 3.5. Statistical Analysis

Data on all the identified GSLs were subjected to multivariate analysis including principal component (PC), orthogonal correction partial least squares discriminant analysis (OPLSDA) and clustering analyses to examine the trends of GSL levels among different accessions using the SIMCA software (V13.3, Umetrics, Sweden). The Pearson correlation analysis was performed to investigate the relations among the GSLs using the SRPLOT statistical software (https://www.bioinformatics.com.cn/login_en, accessed: 4 February 2024).

## 4. Conclusions

In this study, metabolite profiling of glucosinolate content in Chinese cabbage was conducted using the advanced analytical technique Acquity UPLC–MS/MS. The identification and quantification of the compounds revealed metabolic diversity within the Chinese cabbage germplasm. The largest proportion of glucosinolates identified were aliphatic compounds, consistent with previous reports indicating that these are the most prevalent group of glucosinolates in *Brassica* vegetables. The observed variability in glucosinolate content also highlights the significant influence of both genetic and environmental factors, highlighting the importance of using abroad range of germplasms in glucosinolate metabolite analysis. Accessions including IT120044 (Taiwanese landrace), IT186728 (Japanese landrace), IT221789 (Japanese landrace), IT221754 (Japanese landrace), IT278620 (Myanmar landrace), and IT100417 (Taiwanese landrace) were distinctly separated from the rest in the principal component analysis. These accessions exhibited a higher content (above 1000.00 μmol/kg DW) of total glucosinolate as well as higher concentrations of other individual glucosinolates. These accessions represent excellent genetic resources for targeted breeding to enhance total or individual glucosinolate content in Chinese cabbage. The diversity within the germplasm offers the potential for cultivar development. Overall, accessions with a higher GSL content, particularly those with high levels of aliphatic glucosinolates, may offer enhanced nutritional and health benefits due to their potential anticancer properties. Future investigations may target the development of *Brassica* varieties with tailored glucosinolate profiles that align with consumer preferences and the increasing demand for nutritionally enriched vegetables. Additionally, future research may consider evaluating taste profiles across the different Chinese cabbage accessions used in this study.

## Figures and Tables

**Table 1:** Chemical properties of glucosinolates studied in this experiment.

S/N	Trivia Name	Semi-Systematic	Molecular Formula	MW (g/mol)	Precursor Amino Acid	Class
1	Sinigrin	Prop-2-enyl	C_10_H_17_NO_9_S_2_	397.5	Met	Aliphatic
2	Gluconapin	But-3-enyl	C_11_H_19_NO_9_S_2_	373.4	Met	Aliphatic
3	Glucobrassicanapin	Pent-4-enyl	C_12_H_21_NO_9_S_2_	387.4	Met	Aliphatic
4	Epiprogoitrin	(2*S*)-2-Hydroxybut-3-enyl	C_11_H_18_NO_10_S_2_	389.4	Met	Aliphatic
5	Progoitrin	(2*R*)-2-Hydroxybut-3-enyl	C_11_H_19_NO_10_S_2_	389.4	Met	Aliphatic
6	Glucoraphasatin	4-Methylthio-3-butenyl	C_12_H_21_NO_9_S_3_	435.5	Met	Aliphatic
7	Glucoerucin	4-(Methylsulfanyl)butyl, 4-(Methylthio)butyl	C_12_H_23_NO_9_S_3_	421.5	Met	Aliphatic
8	Gluconapoleiferin	(2*S*)-2-Hydroxypent-4-enyl	C_12_H_20_NO_10_S_2_	403.4	Met	Aliphatic
9	Glucoiberin	(*R_S_*)-3-(Methylsulfinyl)propyl	C_11_H_21_NO_10_S_3_	423.5	Met	Aliphatic
10	Glucoraphenin	(*R_S_*,3*E*)-4-(Methylsulfinyl)but-3-enyl	C_12_H_21_NO_10_S_3_	435.5	Met	Aliphatic
11	Glucoraphanin	(*R_S_*)-4-(Methylsulfinyl)butyl	C_12_H_23_NO_10_S_3_	437.5	Met	Aliphatic
12	Glucocheirolin	3-(Methylsulfonyl)propyl	C_11_H_21_NO_11_S_3_	477.6	Met	Aliphatic
13	Glucoalyssin	(*R_S_*)-5-(Methylsulfinyl)pentyl	C_13_H_25_NO_10_S_3_	451.5	Met	Aliphatic
14	Glucotropaeolin	Benzyl	C_14_H_19_NO_9_S_2_	409.4	Phe	Aromatic
15	Gluconasturtiin	2-Phenylethyl, Phenethyl	C_15_H_21_NO_9_S_2_	423.5	Phe	Aromatic
16	Glucobarbarin	(2*S*)-2-hydroxy-2-phenylethyl	C_15_H_21_NO_10_S_2_	477.6	Phe	Aromatic
17	Sinalbin	4-Hydroxybenzyl	C_30_H_42_N_2_O_15_S_2_	425.4	Phe/Tyr	Aromatic
18	4-Hydroxyglucobrassicin	4-Hydroxy-3-indolylmethyl	C_16_H_20_N_2_O_10_S_2_	464.5	Trp	Indole
19	Neoglucobrassicin	1-Methoxyindol-3-ylmethyl, *N*-Methoxyindol-3-ylmethyl	C_17_H_22_N_2_O_10_S_2_	478.5	Trp	Indole
20	4-Methoxyglucobrassicin	4-Methoxyindol-3-ylmethyl	C_17_H_22_N_2_O_10_S_2_	478.5	Trp	Indole

**Table 2:** Mean, median, and range of glucosinolates and antioxidants in Chinese cabbage.

Glucosinolate	Mean	Median	Range
Aliphatic glucosinolates (μmol/kg DW)			
Sinigrin	3.41	2.09	0.27–23.23
Gluconapin	1258.46	642.91	18.07–7397.62
Glucobrassicanapin	1558.49	1265.92	13.43–6460.73
Epiprogoitrin	425.26	301.79	10.35–2412.79
Progoitrin	457.72	356.75	11.33–2026.53
Glucoraphasatin	0.28	0.17	0.00–2.78
Glucoerucin	185.44	14.29	0.03–3918.15
Gluconapoleiferin	222.17	162.15	1.61–931.81
Glucoiberin	0.29	0.19	0.00–2.07
Glucoraphenin	0.44	0.23	0.00–3.46
Glucoraphanin	165.98	61.86	1.14–4242.08
Glucocheirolin	4.34	1.64	0.07–87.44
Glucoalyssin	426.48	256.79	5.10–2147.02
Aromatic glucosinolates (μmol/kg DW)			
Glucotropaeolin	1.56	1.24	0.11–4.91
Gluconasturtiin	283.81	235.01	7.94–1604.40
Glucobarbarin	108.91	93.65	17.74–383.92
Sinalbin	0.08	0.03	0.00–0.31
Indole glucosinolates (μmol/kg DW)			
4-Hydroxyglucobrassicin	61.31	27.81	3.72–796.51
Neoglucobrassicin	253.55	190/45	27.54–1385.02
4-Methoxyglucobrassicin	451.47	353.89	64.11–2016.69
Total glucosinolate	5869.44	4878.99	626.53–21,199.02
Total aliphatic glucosinolate	4708.75	6541.95	149.96–17,972.00
Total aromatic glucosinolate	394.36	12201.62	35.46–1731.84
Total indole glucosinolate	766.32	23493.65	188.14–3261.49

## Data Availability

Data is contained within the article and Supplementary Materials.

## Supplementary Materials

The following supporting information can be downloaded at: https://www.mdpi.com/article/10.3390/ijms25094829/s1.
