# Supplementary material for: Variations of Major Glucosinolates in Diverse Chinese Cabbage (Brassica rapa ssp. pekinensis) Germplasm as Analyzed by UPLC-ESI-MS/MS
Source: Int J Mol Sci. 2024 Apr 29;25(9):4829. doi: 10.3390/ijms25094829 (PMC11084679; doi:10.3390/ijms25094829)
Supplement: Supplementary file 1 [file ijms-25-04829-s001.zip › ijms-2968234-supplementary.pdf]

Variationsin Major Glucosinolatesin Diverse Chinese Cabbage (*Brassica rapa* ssp. *pekinensis)* Germplasm as Analyzed by UPLC-ESI-MS/MS

Seong-Hoon Kim[1,*,†], Kingsley Ochar[1,†],Kanivalan Iwar[1],Yoon-Jung Lee[1],HaeJuKang[2]and Young-Wang Na[1]

[1] National Institute of AgrobiodiversityCenter, National Institute of Agricultural Sciences, Rural Development Administration, Jeonju 5487, Republic of Korea; ocharking@korea.kr (K.O.); kani05@korea.kr (K.I.); yoon112@korea.kr (Y.-J.L.); ywna@korea.kr (Y.-W.N.)

[2] Department of Agrofood Resource, National Institute of Agricultural Sciences, Rural Development Administration, Jeonju 5487, Republic of Korea

* Correspondence: shkim0819@korea.kr

Table S1. Glucosinolates content in the 134 Chinese cabbage cabbage accessions

S/N	IT Number	Accession type	Origin	Sinigrin (mol/kg DW)	Gluconapin (mol/kg DW)	Glucobrassicanapin (mol/kg DW)	Epiprogoitrin (mol/kg DW)	Progoitrin (mol/kg DW)	Glucotropaeolin (mol/kg DW)	Glucoraphasatin (mol/kg DW)
1	100402	landrace	Taiwan	3.11	1830.69	1458.34	98.65	112.27	0.69	0.00
2	100405	landrace	Taiwan	4.05	1729.04	143.27	38.32	43.12	1.55	0.00
3	100417	landrace	Taiwan	5.76	2288.13	1837.84	1563.41	1470.32	0.36	0.16
4	100422	landrace	Taiwan	3.13	522.46	737.67	591.21	632.22	1.05	0.14
5	100437	landrace	Taiwan	6.98	3503.90	1385.26	84.15	90.75	0.75	0.00
6	100439	landrace	Taiwan	3.13	1664.53	2586.18	963.10	949.39	2.47	0.11
7	100451	landrace	Taiwan	2.83	2195.50	3494.91	414.18	429.28	2.62	0.03
8	102914	landrace	China	2.24	582.11	1142.26	156.47	183.48	0.97	0.11
9	112275	landrace	Japan	1.89	851.24	782.28	302.81	321.30	1.23	0.00
10	112664	landrace	China	4.13	2100.08	3254.08	1460.74	1317.22	2.61	0.25
11	112665	landrace	Japan	0.57	54.10	148.30	199.34	221.02	1.22	0.11
12	112667	landrace	Japan	2.22	92.80	704.52	342.64	395.40	0.76	0.00
13	112821	landrace	South Korea	1.22	152.70	1039.51	324.88	357.12	2.77	0.11

14	113557	landrace	South Korea	1.04	266.85	1945.72	418.74	464.46	1.73	0.42
15	120022	landrace	Netherland	4.11	332.80	1223.24	344.07	376.63	1.12	0.00
16	120030	landrace	United States	1.63	75.64	291.83	257.34	287.99	1.00	0.12
17	120034	landrace	Hong Kong	1.16	426.76	946.69	74.17	87.29	0.78	0.15
18	120036	landrace	Hong Kong	7.11	1213.77	2366.53	230.63	563.58	0.49	0.14
19	120040	landrace	Hong Kong	4.84	1934.50	2278.64	1194.13	1175.64	0.39	0.12
20	120042	landrace	HongKong	13.18	1957.43	1881.28	399.20	890.54	0.53	0.13
21	120043	landrace	Taiwan	4.68	1121.94	2157.42	502.31	551.57	0.18	0.00
22	120044	landrace	Taiwan	13.00	5060.19	5987.36	1400.90	1368.44	0.74	0.18
23	120090	landrace	Hongkong	3.09	1073.33	294.07	575.89	632.00	2.08	0.00
24	141425	landrace	South Korea	1.81	109.61	1514.53	192.41	211.50	3.66	0.16
25	160325	landrace	China	9.15	7397.62	3434.83	194.51	206.53	1.23	0.66
26	166986	landrace	China	4.59	2703.40	1694.36	497.96	498.18	0.80	1.07
27	186727	landrace	Japan	2.66	893.80	3137.97	287.86	313.33	2.53	0.15
28	186728	landrace	Japan	9.63	5737.45	296.69	2412.79	1986.40	1.59	0.67
29	186729	landrace	Japan	1.73	600.51	905.58	437.44	459.56	1.29	0.00
30	199696	landrace	Japan	1.95	1311.10	2066.71	300.77	333.94	1.20	0.00
31	199704	landrace	Australia	11.55	445.13	1800.84	619.66	716.01	1.15	0.00
32	199710	landrace	China	5.08	3826.10	3636.10	124.81	154.53	0.32	0.27
33	203324	landrace	Russia	1.52	146.30	1572.34	136.67	181.41	2.06	0.27
34	208851	landrace	South Korea	5.02	848.81	3000.19	414.42	461.60	1.31	0.20
35	212912	landrace	South Korea	0.88	124.46	409.01	220.80	263.00	0.48	0.00
36	214688	landrace	Korea	0.76	110.50	169.94	48.41	60.60	0.75	0.00
37	214998	landrace	South Korea	1.58	360.76	1369.22	43.92	55.14	1.04	0.14
38	214999	landrace	South Korea	7.30	720.23	2628.78	277.73	356.37	2.22	0.00
39	215000	landrace	South Korea	3.65	448.31	794.76	300.43	355.34	2.65	0.52

40	215001	landrace	South Korea	4.22	2521.17	4658.49	198.95	237.56	1.03	0.58
41	215002	landrace	South Korea	3.05	218.67	201.07	81.55	100.84	0.73	0.25
42	215006	landrace	South Korea	2.11	596.41	1681.38	40.76	54.08	3.61	0.00
43	219621	landrace	Brazil	5.34	5283.50	2682.34	739.71	879.59	0.63	1.27
44	221729	landrace	China	3.21	108.48	1020.64	372.10	420.17	0.55	0.00
45	221731	landrace	China	1.44	148.49	1696.89	355.52	436.99	2.32	0.00
46	221732	landrace	China	1.86	685.31	655.99	135.30	192.51	0.53	0.00
47	221733	landrace	China	1.22	24.94	13.43	14.58	18.06	0.71	0.20
48	221734	landrace	China	8.68	751.90	3307.32	220.63	260.68	1.81	0.36
49	221735	landrace	China	4.29	1414.12	2752.73	1318.60	1410.10	2.79	0.00
50	221737	landrace	China	1.36	98.40	694.63	645.60	720.01	1.04	0.16
51	221738	landrace	China	2.17	1346.03	1744.22	280.22	332.15	2.99	0.42
52	221739	landrace	China	2.42	1193.45	2070.63	704.74	815.52	1.26	0.31
53	221741	landrace	China	3.54	490.67	1163.74	143.66	167.91	1.63	0.17
54	221742	landrace	China	2.30	772.02	361.62	861.83	929.87	0.77	0.00
55	221746	landrace	China	2.43	491.10	1251.67	454.11	521.13	0.54	0.00
56	221751	landrace	Japan	10.25	4717.65	1875.72	66.26	81.32	0.41	0.64
57	221752	landrace	Japan	2.09	2150.12	1007.65	809.95	883.32	1.24	0.57
58	221754	landrace	Japan	14.27	5670.40	6460.73	667.63	750.81	1.17	1.65
59	221755	landrace	Japan	4.33	4272.20	3089.45	242.20	286.34	0.98	0.67
60	221760	Cultivar	Taiwan	7.97	577.35	2060.57	895.64	997.56	1.48	0.00
61	221787	landrace	Japan	1.71	779.02	1678.46	465.44	550.45	4.91	0.26
62	221789	landrace	Japan	23.23	7019.61	4952.22	1423.82	1476.41	3.01	2.78
63	227012	landrace	South Korea	4.78	2652.18	4114.99	974.49	1065.69	1.45	2.55
64	227013	landrace	South Korea	1.81	1609.67	1202.90	115.53	134.82	1.94	0.48
65	227906	landrace	Uzbekistan	17.53	2887.56	5926.41	466.43	567.12	1.23	0.94

66	228181	landrace	Japan	0.90	330.62	1040.66	28.22	35.19	4.42	0.04
67	228195	landrace	Peru	6.08	163.42	1788.07	610.28	669.79	3.28	0.00
68	228854	landrace	Uzbekistan	1.73	694.30	2460.65	436.44	488.54	3.70	1.12
69	235343	landrace	South Korea	1.16	222.97	1580.19	576.58	661.48	2.02	0.94
70	235413	landrace	Germany	1.68	436.25	2131.46	382.35	438.73	2.07	0.23
71	235966	landrace	Kyrgyzstan	4.26	1175.26	2760.80	1659.00	1487.13	1.22	0.73
72	247931	landrace	China	1.45	591.57	734.87	293.29	338.82	0.90	0.28
73	247932	Cultivar	China	5.25	2232.25	1471.79	894.87	934.45	1.21	0.08
74	247938	Cultivar	China	1.58	141.24	652.79	347.17	403.46	0.73	0.14
75	247960	landrace	China	1.07	279.20	982.05	534.39	585.18	2.03	0.37
76	247966	Cultivar	China	1.60	305.52	661.14	778.25	905.86	2.96	0.00
77	247979	Cultivar	China	2.00	950.85	1662.55	215.19	241.33	2.72	0.39
78	248400	Cultivar	Japan	14.08	4671.61	1453.30	1575.37	1527.50	1.72	1.18
79	248599	landrace	South Korea	3.26	1920.50	2946.81	392.84	412.89	3.26	0.15
80	251123	Cultivar	China	3.66	2785.41	2612.41	323.50	375.51	3.49	0.00
81	260815	Cultivar	China	10.66	3917.14	4619.06	374.02	414.86	0.69	0.45
82	278620	landrace	Myanmar	3.01	1908.45	1117.54	2107.32	2026.53	0.28	0.44
83	278640	landrace	South Korea	1.84	692.90	1474.02	228.52	260.34	1.35	0.00
84	278703	landrace	South Korea	0.34	142.27	204.60	116.09	136.84	1.64	0.00
85	293003	landrace	Mongolia	2.64	991.90	1002.55	602.45	693.08	1.92	0.48
86	293004	landrace	Mongolia	2.53	1749.07	2141.87	527.57	543.52	1.02	0.31
87	293076	landrace	Austria	0.61	534.81	861.35	103.34	119.88	1.54	0.00
88	321040	landrace	South Korea	1.52	573.86	952.35	155.67	177.43	0.40	0.20
89	321041	landrace	South Korea	0.48	41.00	232.94	63.67	73.34	1.44	0.13
90	327479	Cultivar	China	3.95	404.14	2116.94	361.63	391.85	0.87	0.15
91	336315	landrace	South Korea	0.48	43.09	145.57	162.15	182.56	1.45	0.40

92	339601	Cultivar	South Korea	2.12	1642.25	1847.31	655.74	696.02	1.09	0.41
93	339602	landrace	South Korea	0.51	132.01	194.86	324.60	366.50	0.39	0.19
94	344732	landrace	Malaysia	1.44	1761.53	494.63	33.47	41.75	0.33	0.21
95	344733	landrace	Japan	0.27	459.26	412.52	95.35	108.14	1.16	0.14
96	344734	landrace	Japan	2.85	318.83	1418.15	365.23	411.87	0.52	0.26
97	344735	landrace	Newzealand	5.02	308.10	893.59	645.47	680.12	0.93	0.13
98	344736	landrace	Austria	1.76	89.82	584.95	249.78	280.11	3.65	0.15
99	344737	landrace	Austria	1.68	435.57	1255.26	784.48	789.85	0.61	0.32
100	344740	landrace	France	2.82	970.35	1247.29	340.30	391.23	4.55	0.44
101	344741	landrace	Netherland	0.69	143.83	514.67	149.53	165.19	1.99	0.00
102	344742	landrace	United Kingdom	1.76	886.31	1268.27	1052.70	1064.81	0.96	0.00
103	344743	Cultivar	Russia	2.62	2138.87	3217.83	252.84	288.51	2.12	0.39
104	344746	Cultivar	China	3.67	6513.62	1533.14	288.71	308.86	0.72	0.35
105	344747	Cultivar	China	1.17	1508.96	793.42	437.74	448.83	1.20	0.38
106	344748	Cultivar	China	0.85	262.06	529.84	60.03	76.18	3.90	0.00
107	344749	Cultivar	China	2.15	75.81	926.57	184.86	208.73	2.96	0.31
108	344750	Cultivar	China	2.10	242.70	1380.36	126.67	137.14	4.65	0.00
109	344751	Cultivar	Russia	1.21	29.23	171.41	257.55	295.52	3.19	0.00
110	344752	landrace	China	0.56	18.07	209.23	148.68	166.91	2.17	0.22
111	344755	landrace	Japan	0.89	1050.38	1263.56	15.40	18.13	1.27	0.52
112	344756	landrace	China	1.53	52.43	184.02	237.05	270.54	3.37	0.17
113	344757	Cultivar	South Korea	0.67	85.94	452.95	633.05	663.12	1.01	0.00
114	344758	Cultivar	South Korea	0.65	1046.48	1768.34	27.10	32.37	1.84	0.00
115	344759	Cultivar	South Korea	1.92	1033.07	1599.84	931.82	979.43	1.94	0.35
116	344760	Cultivar	South Korea	1.44	372.60	1528.19	905.69	921.91	0.85	0.55

117	344761	Genetic material	South Korea	0.53	68.99	249.18	85.65	99.05	1.26	0.00
118	344763	landrace	China	0.52	199.16	212.46	129.73	156.07	0.80	0.00
119	344764	Cultivar	South Korea	0.32	33.46	20.86	10.35	11.33	1.61	0.28
120	344765	Cultivar	South Korea	4.16	1417.70	514.15	82.72	97.49	2.05	0.19
121	344766	Cultivar	South Korea	0.95	291.09	1104.90	16.50	19.03	1.64	0.41
122	344767	Cultivar	South Korea	2.01	1512.65	983.72	112.05	131.53	0.11	0.13
123	344768	Cultivar	South Korea	11.63	3755.49	2351.99	366.60	415.87	0.23	0.40
124	344770	Cultivar	South Korea	1.37	239.58	549.42	107.58	123.21	0.38	0.00
125	344771	landrace	China	0.55	120.74	453.42	184.06	213.73	1.41	0.19
126	344772	landrace	China	2.65	1829.43	1940.85	510.74	541.67	1.29	0.22
127	344773	landrace	China	1.01	223.93	514.05	97.76	120.47	0.94	0.00
128	344774	landrace	China	1.15	159.40	852.06	348.44	383.74	1.53	0.18
129	344775	landrace	Thailand	0.77	300.25	729.03	27.76	34.41	1.73	0.33
130	344776	landrace	Thailand	1.26	345.31	1173.17	120.18	143.48	0.89	0.11
131	344777	landrace	Thailand	1.62	1946.12	2408.90	334.92	382.22	1.00	0.47
132	344778	landrace	South Korea	2.01	65.33	975.73	130.67	152.58	0.77	0.17
133	344779	landrace	South Korea	0.74	493.17	1614.43	42.48	49.34	2.43	0.24
134	344780	landrace	South Korea	1.46	859.02	1845.72	79.12	95.29	0.62	0.37

Table S1. Glucosinolates content in the 134 Chinese cabbage cabbage accessions (Cont'd)

S/N	IT Number	Accession type	Origin	Glucoerucin (mol/kg DW)	Gluconasturtiin (mol/kg DW)	Glucobarbarin (mol/kg DW)	Gluconapoleiferin (mol/kg DW)	Glucoiberin (mol/kg DW)	Sinalbin (mol/kg DW)	Glucoraphenin (mol/kg DW)
1	100402	landrace	Taiwan	0.39	190.81	126.40	24.47	0.18	0.25	0.10
2	100405	landrace	Taiwan	11.16	393.77	148.02	1.61	0.69	0.00	0.03

3	100417	landrace	Taiwan	1812.24	840.72	49.48	502.31	0.72	0.01	1.14
4	100422	landrace	Taiwan	855.44	531.95	90.65	271.93	0.43	0.22	0.20
5	100437	landrace	Taiwan	0.48	169.81	88.78	6.98	0.24	0.02	0.17
6	100439	landrace	Taiwan	55.45	145.04	105.90	358.93	0.21	0.24	0.36
7	100451	landrace	Taiwan	1.12	302.04	237.84	107.26	0.20	0.23	0.58
8	102914	landrace	China	3.83	87.41	56.54	53.56	0.23	0.23	0.33
9	112275	landrace	Japan	14.96	301.56	97.64	82.97	0.25	0.24	0.85
10	112664	landrace	China	55.13	550.10	136.29	638.28	0.24	0.26	1.61
11	112665	landrace	Japan	178.28	385.53	19.43	179.41	0.24	0.29	0.08
12	112667	landrace	Japan	204.36	615.18	65.21	330.24	0.35	0.24	0.07
13	112821	landrace	South Korea	0.70	39.24	89.57	465.45	0.18	0.24	0.03
14	113557	landrace	South Korea	0.51	57.65	139.25	844.90	0.18	0.02	0.08
15	120022	landrace	Netherland	648.00	527.32	99.28	323.57	0.96	0.24	0.09
16	120030	landrace	United States	127.87	372.23	191.04	264.20	0.30	0.02	0.09
17	120034	landrace	Hong Kong	7.37	88.51	80.67	26.82	1.34	0.24	0.28
18	120036	landrace	Hong Kong	356.17	518.36	91.68	133.27	0.90	0.23	0.20
19	120040	landrace	Hong Kong	1062.39	915.99	72.06	290.94	0.44	0.21	0.47
20	120042	landrace	Hong Kong	1362.38	479.72	80.57	425.75	1.47	0.00	0.22
21	120043	landrace	Taiwan	309.54	1154.83	90.34	217.57	0.45	0.25	0.32
22	120044	landrace	Taiwan	978.56	1604.40	126.70	499.72	1.76	0.00	1.75
23	120090	landrace	Hongkong	611.77	361.38	36.25	30.94	0.40	0.24	0.11
24	141425	landrace	South Korea	0.46	231.14	138.60	458.53	0.20	0.31	0.10
25	160325	landrace	China	3.18	499.76	159.43	10.35	0.66	0.00	1.37
26	166986	landrace	China	1037.20	491.45	93.78	36.62	0.84	0.26	3.32
27	186727	landrace	Japan	2.17	307.10	117.93	160.82	0.24	0.22	0.23

28	186728	landrace	Japan	1685.23	265.74	136.98	35.49	2.07	0.31	3.46
29	186729	landrace	Japan	37.11	387.30	62.36	127.56	0.21	0.00	1.26
30	199696	landrace	Japan	3.57	246.96	100.92	53.10	0.23	0.00	0.90
31	199704	landrace	Australia	18.38	443.70	158.61	599.91	1.22	0.03	0.18
32	199710	landrace	China	1.21	212.79	113.23	14.67	0.24	0.02	0.23
33	203324	landrace	Russia	0.69	48.79	98.03	638.31	0.23	0.24	0.14
34	208851	landrace	South Korea	0.03	359.20	194.15	242.16	0.23	0.05	0.15
35	212912	landrace	South Korea	48.66	190.91	184.32	227.69	0.35	0.24	0.13
36	214688	landrace	Korea	0.72	39.81	91.70	47.34	0.27	0.24	0.13
37	214998	landrace	South Korea	0.12	25.07	17.74	17.68	0.18	0.02	0.07
38	214999	landrace	South Korea	0.62	172.16	105.49	326.01	0.40	0.04	0.11
39	215000	landrace	South Korea	3.11	13.22	92.31	125.48	0.35	0.00	0.10
40	215001	landrace	South Korea	206.06	441.22	112.84	56.70	0.36	0.25	1.71
41	215002	landrace	South Korea	5.66	72.40	81.54	17.82	0.28	0.23	0.09
42	215006	landrace	South Korea	7.66	65.95	94.10	19.92	0.30	0.03	0.15
43	219621	landrace	Brazil	6.31	74.98	95.07	123.54	0.22	0.03	0.31
44	221729	landrace	China	119.83	238.88	51.01	505.14	0.44	0.03	0.08
45	221731	landrace	China	3.47	130.57	118.35	812.36	0.23	0.23	0.24
46	221732	landrace	China	16.79	167.26	109.35	25.01	0.24	0.26	0.35
47	221733	landrace	China	28.33	18.64	42.51	3.61	0.20	0.04	0.03
48	221734	landrace	China	0.34	47.25	102.12	325.94	0.18	0.23	0.26
49	221735	landrace	China	4.30	695.93	190.31	658.65	0.18	0.23	1.10
50	221737	landrace	China	131.47	507.44	45.14	626.95	0.37	0.22	0.27
51	221738	landrace	China	10.94	207.11	184.63	281.98	0.19	0.04	0.44
52	221739	landrace	China	74.52	273.98	245.22	241.76	0.27	0.21	0.85
53	221741	landrace	China	176.82	230.16	151.20	41.51	0.48	0.02	0.24

54	221742	landrace	China	1319.96	362.41	40.90	119.38	0.69	0.04	0.17
55	221746	landrace	China	10.47	443.26	249.91	347.39	0.19	0.25	0.08
56	221751	landrace	Japan	10.68	489.20	253.16	2.50	0.50	0.25	0.46
57	221752	landrace	Japan	74.11	334.84	170.36	81.53	0.30	0.29	0.50
58	221754	landrace	Japan	57.74	397.51	197.85	218.24	0.46	0.24	0.87
59	221755	landrace	Japan	1.77	155.29	204.57	12.87	0.21	0.00	0.46
60	221760	Cultivar	Taiwan	68.62	491.64	119.18	578.57	0.78	0.21	0.28
61	221787	landrace	Japan	8.10	280.00	171.11	81.35	0.08	0.00	0.14
62	221789	landrace	Japan	2.33	667.36	272.01	141.17	0.19	0.00	0.29
63	227012	landrace	South Korea	0.20	277.40	324.96	373.42	0.07	0.00	0.09
64	227013	landrace	South Korea	27.27	155.62	93.51	11.56	0.11	0.02	0.10
65	227906	landrace	Uzbekistan	0.66	194.26	183.35	303.15	0.10	0.02	0.10
66	228181	landrace	Japan	0.67	24.92	63.33	10.16	0.07	0.03	0.03
67	228195	landrace	Peru	141.82	520.45	304.08	478.64	0.36	0.06	0.20
68	228854	landrace	Uzbekistan	1.93	161.83	150.18	401.68	0.07	0.01	0.10
69	235343	landrace	South Korea	1.70	83.55	167.41	559.75	0.08	0.04	0.04
70	235413	landrace	Germany	23.36	414.38	275.65	248.80	0.10	0.00	0.16
71	235966	landrace	Kyrgyzstan	68.93	357.60	193.70	496.65	0.13	0.03	0.63
72	247931	landrace	China	572.59	140.95	45.32	77.35	0.10	0.04	0.12
73	247932	Cultivar	China	456.49	354.77	59.27	134.53	0.21	0.01	0.27
74	247938	Cultivar	China	8.86	115.57	126.39	243.68	0.09	0.00	0.12
75	247960	landrace	China	24.87	132.72	103.04	357.52	0.12	0.02	0.14
76	247966	Cultivar	China	49.29	288.61	111.79	334.65	0.13	0.00	0.23
77	247979	Cultivar	China	9.11	76.07	136.88	63.77	0.07	0.04	0.28
78	248400	Cultivar	Japan	1.16	31.00	59.21	78.99	0.07	0.03	0.37
79	248599	landrace	South Korea	0.62	447.45	184.73	141.90	0.07	0.03	0.18

80	251123	Cultivar	China	0.64	144.53	35.66	86.14	0.14	0.03	0.58
81	260815	Cultivar	China	0.58	473.86	383.92	183.59	0.13	0.03	0.19
82	278620	landrace	Myanmar	3918.15	728.66	59.90	168.61	0.11	0.00	0.99
83	278640	landrace	South Korea	2.45	64.13	129.41	96.31	0.09	0.00	0.05
84	278703	landrace	South Korea	29.67	51.30	71.14	19.80	0.07	0.01	0.11
85	293003	landrace	Mongolia	109.58	296.24	195.71	140.08	0.11	0.05	0.13
86	293004	landrace	Mongolia	0.76	193.31	163.88	268.17	0.07	0.03	0.68
87	293076	landrace	Austria	0.66	228.20	40.43	35.94	0.09	0.00	0.18
88	321040	landrace	South Korea	0.11	101.87	106.70	143.44	0.08	0.00	0.19
89	321041	landrace	South Korea	0.23	18.10	48.55	175.91	0.08	0.00	0.05
90	327479	Cultivar	China	3.32	427.69	54.07	460.08	0.12	0.00	0.03
91	336315	landrace	South Korea	3.16	11.01	36.06	188.33	0.00	0.00	0.00
92	339601	Cultivar	South Korea	315.58	574.49	63.81	237.83	0.00	0.01	1.58
93	339602	landrace	South Korea	10.21	23.76	48.11	213.42	0.02	0.00	0.41
94	344732	landrace	Malaysia	186.88	491.27	124.74	1.73	0.19	0.01	0.25
95	344733	landrace	Japan	50.84	102.17	61.05	18.92	0.02	0.01	0.25
96	344734	landrace	Japan	24.44	82.45	81.81	358.89	0.09	0.09	0.19
97	344735	landrace	Newzealand	99.49	516.91	99.51	389.29	0.70	0.02	0.42
98	344736	landrace	Austria	71.25	204.38	81.37	416.58	0.03	0.01	0.23
99	344737	landrace	Austria	0.67	263.69	119.61	474.27	0.00	0.02	0.47
100	344740	landrace	France	2000.28	359.01	46.69	88.15	0.24	0.01	0.77
101	344741	landrace	Netherland	99.99	79.37	31.10	168.34	0.01	0.02	0.31
102	344742	landrace	United Kingdom	72.15	631.45	66.04	462.39	0.01	0.08	1.16
103	344743	Cultivar	Russia	4.12	178.12	163.76	150.90	0.00	0.00	0.16
104	344746	Cultivar	China	189.18	247.85	87.23	14.65	0.49	0.07	3.43

105	344747	Cultivar	China	13.61	35.37	83.10	116.76	0.07	0.01	0.36
106	344748	Cultivar	China	0.11	30.04	105.14	115.67	0.00	0.00	0.17
107	344749	Cultivar	China	0.66	89.46	91.24	547.02	0.08	0.00	0.24
108	344750	Cultivar	China	5.22	172.17	36.15	176.23	0.13	0.01	0.18
109	344751	Cultivar	Russia	183.23	254.72	138.73	206.96	0.29	0.03	0.15
110	344752	landrace	China	1.60	44.00	55.72	543.34	0.00	0.00	0.15
111	344755	landrace	Japan	0.33	20.83	52.36	4.07	0.00	0.01	0.21
112	344756	landrace	China	210.21	273.16	127.71	224.85	1.13	0.03	0.21
113	344757	Cultivar	South Korea	240.00	438.75	142.20	627.02	0.08	0.01	0.59
114	344758	Cultivar	South Korea	1.01	157.83	32.95	5.18	0.00	0.04	0.54
115	344759	Cultivar	South Korea	476.16	438.76	61.79	326.84	0.24	0.00	1.13
116	344760	Cultivar	South Korea	33.14	572.36	166.73	931.81	0.06	0.01	0.60
117	344761	Genetic material	South Korea	54.58	264.37	80.37	163.48	0.08	0.00	0.14
118	344763	landrace	China	89.82	60.42	68.45	51.12	0.00	0.01	0.37
119	344764	Cultivar	South Korea	2.04	7.94	25.89	7.61	0.00	0.02	0.12
120	344765	Cultivar	South Korea	0.79	49.46	34.69	12.46	0.12	0.00	0.34
121	344766	Cultivar	South Korea	0.15	62.16	57.29	20.47	0.01	0.09	0.08
122	344767	Cultivar	South Korea	86.17	271.58	80.24	28.25	0.33	0.04	0.58
123	344768	Cultivar	South Korea	495.04	556.69	62.90	76.72	1.93	0.02	0.85
124	344770	Cultivar	South Korea	210.22	550.82	23.73	65.99	0.28	0.02	0.27
125	344771	landrace	China	5.02	129.34	39.92	219.15	0.05	0.00	0.53
126	344772	landrace	China	69.21	747.54	80.62	168.04	0.48	0.04	1.52
127	344773	landrace	China	49.50	69.76	47.73	55.27	0.18	0.03	0.29
128	344774	landrace	China	59.26	469.40	64.07	280.99	0.49	0.00	0.60
129	344775	landrace	Thailand	2.70	40.94	38.35	28.90	0.08	0.00	0.23

130	344776	landrace	Thailand	19.60	61.51	59.68	53.33	0.10	0.03	0.92
131	344777	landrace	Thailand	107.70	243.41	102.07	140.63	0.04	0.03	0.91
132	344778	landrace	South Korea	0.73	49.84	59.22	695.04	0.03	0.05	0.01
133	344779	landrace	South Korea	0.03	157.62	52.05	40.71	0.00	0.00	0.28
134	344780	landrace	South Korea	0.07	76.80	52.87	32.12	0.02	0.01	0.13

Table S1. Glucosinolates content in the 134 Chinese cabbage cabbage accessions (Cont'd)

S/N	IT Number	Accession type	Origin	Glucoraphanin (mol/kg DW)	Glucocheirolin (mol/kg DW)	Glucoalyssin (mol/kg DW)	4-Hydroxyglucobrassicin (mol/kg DW)	Neoglucobrassicin (mol/kg DW)	4-Methoxyglucobrassicin (mol/kg DW)	Total glucosinolate (mol/kg DW)
1	100402	landrace	Taiwan	23.23	0.80	82.42	17.50	143.05	64.11	4177.45
2	100405	landrace	Taiwan	368.59	12.30	292.02	38.37	438.93	189.91	3854.74
3	100417	landrace	Taiwan	873.16	29.85	1419.28	35.01	115.40	1024.37	13869.67
4	100422	landrace	Taiwan	508.04	16.96	1227.79	16.04	245.97	751.64	7005.12
5	100437	landrace	Taiwan	21.64	0.44	78.27	19.84	201.25	149.73	5809.44
6	100439	landrace	Taiwan	72.55	2.25	231.85	308.68	216.82	413.97	8081.16
7	100451	landrace	Taiwan	4.15	0.26	38.18	39.98	193.54	507.91	7972.64
8	102914	landrace	China	23.11	0.72	140.65	10.78	107.86	409.55	2962.43
9	112275	landrace	Japan	92.38	1.65	192.10	147.13	47.02	312.96	3552.50
10	112664	landrace	China	323.55	7.79	1081.42	432.50	320.53	573.47	12260.28
11	112665	landrace	Japan	139.44	2.81	499.14	29.61	100.38	344.22	2503.52
12	112667	landrace	Japan	129.30	3.21	562.77	172.62	190.70	1187.40	4999.98
13	112821	landrace	South Korea	17.07	0.37	79.11	19.00	805.82	451.04	3846.15
14	113557	landrace	South Korea	12.76	0.28	53.58	98.66	543.84	288.48	5139.14
15	120022	landrace	Netherland	482.34	9.87	1521.71	244.88	450.15	1112.16	7702.51
16	120030	landrace	United States	213.05	4.19	880.28	229.02	663.50	327.76	4189.10
17	120034	landrace	Hong Kong	481.95	9.59	370.39	15.51	87.42	262.05	2969.15

18	120036	landrace	Hong Kong	214.64	4.46	650.92	11.21	141.32	704.96	7210.56
19	120040	landrace	Hong Kong	592.53	12.91	1543.68	12.01	126.51	817.88	12036.27
20	120042	landrace	Hong Kong	456.91	10.93	1582.32	16.67	261.28	775.64	10596.15
21	120043	landrace	Taiwan	111.78	2.51	597.07	126.58	150.44	954.12	8053.91
22	120044	landrace	Taiwan	499.89	13.23	2147.02	18.96	271.10	1205.12	21199.02
23	120090	landrace	Hongkong	408.92	8.60	672.32	24.34	189.83	668.00	5593.56
24	141425	landrace	South Korea	24.89	0.47	130.96	63.78	1116.42	270.25	4469.77
25	160325	landrace	China	81.41	2.06	147.70	31.16	347.01	179.08	12707.70
26	166986	landrace	China	338.41	6.90	189.97	24.54	125.95	126.57	7876.17
27	186727	landrace	Japan	15.16	0.40	180.27	329.81	53.74	550.45	6356.84
28	186728	landrace	Japan	4242.08	87.44	244.69	23.80	190.19	646.66	18009.37
29	186729	landrace	Japan	133.83	2.55	171.57	33.54	65.48	610.10	4038.99
30	199696	landrace	Japan	19.40	0.52	103.50	36.14	220.65	687.40	5488.98
31	199704	landrace	Australia	90.12	1.29	904.98	122.52	246.20	319.65	6501.13
32	199710	landrace	China	11.90	0.25	33.00	268.80	78.25	207.58	8689.35
33	203324	landrace	Russia	11.62	0.38	54.53	22.56	364.07	192.25	3472.41
34	208851	landrace	South Korea	12.00	0.40	26.10	26.54	668.23	243.51	6504.31
35	212912	landrace	South Korea	86.21	1.63	275.31	59.35	150.83	644.16	2888.43
36	214688	landrace	Korea	31.74	0.57	87.58	5.51	93.37	127.55	917.50
37	214998	landrace	South Korea	3.36	0.07	16.31	26.55	83.29	147.79	2170.04
38	214999	landrace	South Korea	28.53	0.26	171.20	16.45	226.91	233.28	5274.09
39	215000	landrace	South Korea	142.19	2.54	593.64	34.48	225.29	404.87	3543.20
40	215001	landrace	South Korea	43.00	0.95	70.80	46.22	372.00	160.34	9134.44
41	215002	landrace	South Korea	126.85	1.88	313.42	276.70	105.92	211.02	1819.95
42	215006	landrace	South Korea	22.99	0.59	380.37	30.70	257.34	636.78	3895.25
43	219621	landrace	Brazil	207.39	3.98	154.11	28.66	189.28	274.53	10750.76
44	221729	landrace	China	51.13	1.55	297.01	30.50	236.06	582.94	4039.75

45	221731	landrace	China	24.22	0.48	283.73	14.96	295.92	430.91	4757.30
46	221732	landrace	China	183.94	5.13	540.54	43.87	139.17	295.89	3199.30
47	221733	landrace	China	46.27	1.32	92.22	20.12	183.12	180.06	689.61
48	221734	landrace	China	3.07	0.35	150.95	51.24	375.03	300.34	5908.67
49	221735	landrace	China	68.87	2.20	530.07	37.01	372.90	384.80	9849.19
50	221737	landrace	China	145.43	4.14	1045.96	110.30	273.91	709.66	5762.47
51	221738	landrace	China	44.37	1.67	192.14	79.85	174.14	653.63	5539.32
52	221739	landrace	China	151.83	5.48	394.99	97.63	152.02	621.77	7048.87
53	221741	landrace	China	74.17	3.29	735.20	10.61	43.03	331.13	3769.19
54	221742	landrace	China	598.63	27.30	916.92	39.93	33.02	840.78	7228.52
55	221746	landrace	China	29.10	1.40	297.52	15.88	51.25	190.29	4357.97
56	221751	landrace	Japan	33.08	1.07	1147.41	21.80	87.37	131.57	8931.29
57	221752	landrace	Japan	276.50	12.96	513.49	60.23	82.33	159.88	6622.27
58	221754	landrace	Japan	181.75	7.77	424.86	100.41	100.89	294.29	15549.54
59	221755	landrace	Japan	37.98	1.31	242.79	96.35	85.22	173.84	8908.85
60	221760	Cultivar	Taiwan	62.74	2.65	866.74	20.98	120.57	794.77	7668.30
61	221787	landrace	Japan	17.46	0.66	217.81	22.67	129.90	550.33	4959.85
62	221789	landrace	Japan	22.26	1.06	121.90	12.11	240.84	297.48	16680.10
63	227012	landrace	South Korea	1.32	0.14	7.06	21.65	247.92	411.51	10481.89
64	227013	landrace	South Korea	43.78	2.15	173.38	12.78	84.19	246.63	3918.24
65	227906	landrace	Uzbekistan	4.85	0.49	77.16	192.61	63.61	199.19	11086.76
66	228181	landrace	Japan	1.14	0.18	12.26	9.31	59.05	311.07	1932.28
67	228195	landrace	Peru	130.46	6.05	1062.92	119.66	1088.22	111.04	7204.90
68	228854	landrace	Uzbekistan	20.39	0.83	101.92	22.48	172.30	153.87	5274.08
69	235343	landrace	South Korea	12.85	0.39	35.73	63.16	374.03	85.80	4429.86
70	235413	landrace	Germany	23.83	0.33	337.10	40.52	176.27	356.19	5289.47
71	235966	landrace	Kyrgyzstan	114.49	4.82	531.92	10.33	154.41	339.67	9361.69

72	247931	landrace	China	125.10	5.00	401.15	12.21	59.47	356.56	3757.14
73	247932	Cultivar	China	185.31	8.02	885.83	3.72	96.91	430.34	8155.58
74	247938	Cultivar	China	15.69	0.62	130.11	28.18	45.66	191.50	2453.56
75	247960	landrace	China	23.30	0.81	217.78	12.73	355.03	320.24	3932.62
76	247966	Cultivar	China	54.78	1.58	364.62	15.89	128.74	503.75	4509.39
77	247979	Cultivar	China	43.40	1.96	100.36	41.01	232.00	274.70	4054.70
78	248400	Cultivar	Japan	9.53	0.35	5.10	33.43	66.07	209.86	9739.93
79	248599	landrace	South Korea	3.97	0.19	10.83	11.66	364.76	176.92	7023.03
80	251123	Cultivar	China	60.97	1.81	403.89	32.94	553.47	140.24	7565.01
81	260815	Cultivar	China	10.37	0.37	30.46	25.98	319.18	113.81	10879.35
82	278620	landrace	Myanmar	437.83	14.46	1002.28	170.25	34.04	308.68	14007.51
83	278640	landrace	South Korea	22.03	0.54	83.14	16.41	125.91	165.14	3364.57
84	278703	landrace	South Korea	34.85	0.78	101.93	6.56	57.42	283.89	1259.30
85	293003	landrace	Mongolia	169.73	4.46	550.31	23.85	200.71	426.35	5412.34
86	293004	landrace	Mongolia	29.67	0.75	155.28	39.87	236.28	406.19	6460.83
87	293076	landrace	Austria	5.87	0.27	51.02	7.34	191.45	527.39	2710.37
88	321040	landrace	South Korea	7.80	0.41	10.66	7.61	154.69	92.03	2487.04
89	321041	landrace	South Korea	9.01	0.27	13.11	9.98	743.17	242.57	1674.02
90	327479	Cultivar	China	9.61	0.31	149.40	16.58	90.42	582.37	5073.52
91	336315	landrace	South Korea	28.83	0.82	78.94	18.80	220.23	310.66	1432.55
92	339601	Cultivar	South Korea	95.20	3.00	318.66	20.45	253.46	738.79	7467.80
93	339602	landrace	South Korea	75.15	1.84	162.65	9.93	27.54	196.20	1788.30
94	344732	landrace	Malaysia	787.60	18.94	589.75	6.65	38.07	143.42	4722.85
95	344733	landrace	Japan	119.23	3.02	231.64	10.20	264.94	543.99	2483.11
96	344734	landrace	Japan	110.94	2.22	1150.11	23.25	53.79	322.42	4728.39
97	344735	landrace	Newzealand	173.60	3.62	1318.12	223.98	659.68	526.24	6544.94
98	344736	landrace	Austria	71.03	1.55	729.85	19.59	1385.02	346.17	4537.28

99	344737	landrace	Austria	10.59	0.25	64.13	43.66	113.12	439.87	4798.12
100	344740	landrace	France	464.67	13.47	2023.80	796.51	448.30	2016.69	11215.58
101	344741	landrace	Netherland	89.07	2.14	484.38	25.44	262.95	872.66	3091.68
102	344742	landrace	United Kingdom	190.77	3.85	440.00	67.35	216.84	777.45	7204.37
103	344743	Cultivar	Russia	8.87	0.18	190.07	9.84	191.20	462.68	7263.08
104	344746	Cultivar	China	568.60	14.79	525.59	30.53	105.69	131.42	10568.57
105	344747	Cultivar	China	218.24	5.12	107.84	32.77	576.56	170.54	4552.04
106	344748	Cultivar	China	12.41	0.27	31.34	18.76	499.87	204.98	1951.64
107	344749	Cultivar	China	34.77	0.78	436.03	33.23	727.65	386.06	3748.61
108	344750	Cultivar	China	8.42	0.17	201.71	10.12	117.91	318.42	2940.46
109	344751	Cultivar	Russia	108.37	2.68	831.87	78.68	739.27	541.22	3844.31
110	344752	landrace	China	43.66	1.44	198.83	16.41	123.42	236.67	1811.09
111	344755	landrace	Japan	13.01	0.25	29.50	21.68	80.10	384.12	2956.63
112	344756	landrace	China	442.96	11.58	1221.90	48.60	622.45	354.94	4288.84
113	344757	Cultivar	South Korea	331.58	7.24	854.49	187.46	143.42	261.47	5071.04
114	344758	Cultivar	South Korea	8.31	0.18	25.43	5.57	195.15	394.10	3703.08
115	344759	Cultivar	South Korea	622.39	17.18	1223.67	9.90	149.57	1038.65	8914.63
116	344760	Cultivar	South Korea	68.17	1.43	511.50	166.97	186.31	779.55	7149.87
117	344761	Genetic material	South Korea	43.00	0.42	220.73	25.35	205.16	497.11	2059.46
118	344763	landrace	China	171.47	3.90	306.03	13.04	116.58	492.67	2072.60
119	344764	Cultivar	South Korea	33.63	0.86	29.09	18.45	252.26	170.40	626.53
120	344765	Cultivar	South Korea	27.64	1.03	32.15	24.45	497.95	368.93	3168.45
121	344766	Cultivar	South Korea	1.44	0.09	10.82	14.80	137.70	589.83	2329.44
122	344767	Cultivar	South Korea	316.87	7.04	500.91	18.92	92.37	339.33	4484.83
123	344768	Cultivar	South Korea	407.48	11.05	1632.50	123.07	122.31	1417.95	11810.73
124	344770	Cultivar	South Korea	289.88	7.76	790.31	36.26	87.65	639.56	3724.29

125	344771	landrace	China	20.17	0.62	172.69	45.92	311.99	932.72	2852.22
126	344772	landrace	China	140.13	3.75	477.41	31.83	238.70	1208.22	7994.34
127	344773	landrace	China	137.23	3.37	480.32	27.43	69.20	352.83	2251.29
128	344774	landrace	China	97.34	2.26	825.19	118.76	339.11	1450.30	5454.28
129	344775	landrace	Thailand	28.29	0.78	149.61	19.87	213.03	334.44	1951.50
130	344776	landrace	Thailand	102.52	2.55	542.11	32.61	78.52	599.62	3337.48
131	344777	landrace	Thailand	247.59	5.88	453.86	38.10	970.83	875.28	8261.56
132	344778	landrace	South Korea	36.12	0.60	268.88	136.39	219.08	130.40	2923.66
133	344779	landrace	South Korea	4.61	0.09	25.92	29.78	352.74	768.23	3634.89
134	344780	landrace	South Korea	21.00	0.58	35.16	36.54	422.20	167.03	3726.14

Table S2. List of Chinese cabbage accessions used in the study and their origin

S/N	IT Number	Accession Type	Origin	Abbereviation
1	IT100402	landrace	Taiwan	TWN
2	IT100405	landrace	Taiwan	TWN
3	IT100417	landrace	Taiwan	TWN
4	IT100422	landrace	Taiwan	TWN
5	IT100437	landrace	Taiwan	TWN
6	IT100439	landrace	Taiwan	TWN
7	IT100451	landrace	Taiwan	TWN
8	IT102914	landrace	China	CHN
8	IT112275	landrace	Japan	JPN
10	IT112664	landrace	China	CHN
11	IT112665	landrace	Japan	JPN
12	IT112667	landrace	Japan	JPN
13	IT112821	landrace	South Korea	KOR
14	IT113557	landrace	South Korea	KOR
15	IT120022	landrace	Netherland	NLD
16	IT120030	landrace	United States	USA
17	IT120034	landrace	Hong Kong	HKG

18	IT120036	landrace	Hong Kong	HKG
19	IT120040	landrace	Hong Kong	HKG
20	IT120042	landrace	Hong Kong	HKG
21	IT120043	landrace	Taiwan	TWN
22	IT120044	landrace	Taiwan	TWN
23	IT120090	landrace	Hongkong	HKG
24	IT141425	landrace	South Korea	KOR
25	IT160325	landrace	China	CHN
26	IT166986	landrace	China	CHN
27	IT186727	landrace	Japan	JPN
28	IT186728	landrace	Japan	JPN
29	IT186729	landrace	Japan	JPN
30	IT199696	landrace	Japan	JPN
31	IT199704	landrace	Australia	AUS
32	IT199710	landrace	China	CHN
33	IT203324	landrace	Russia	RUS
34	IT208851	landrace	South Korea	KOR
35	IT212912	landrace	South Korea	KOR
36	IT214688	landrace	Korea	PRK
37	IT214998	landrace	South Korea	KOR
38	IT214999	landrace	South Korea	KOR
39	IT215000	landrace	South Korea	KOR
40	IT215001	landrace	South Korea	KOR
41	IT215002	landrace	South Korea	KOR
42	IT215006	landrace	South Korea	KOR
43	IT219621	landrace	Brazil	BRA
44	IT221729	landrace	China	CHN
45	IT221731	landrace	China	CHN
46	IT221732	landrace	China	CHN
47	IT221733	landrace	China	CHN
48	IT221734	landrace	China	CHN
49	IT221735	landrace	China	CHN
50	IT221737	landrace	China	CHN

51	IT221738	landrace	China	CHN
52	IT221739	landrace	China	CHN
53	IT221741	landrace	China	CHN
54	IT221742	landrace	China	CHN
55	IT221746	landrace	China	CHN
56	IT221751	landrace	Japan	JPN
57	IT221752	landrace	Japan	JPN
58	IT221754	landrace	Japan	JPN
59	IT221755	landrace	Japan	JPN
60	IT221760	Cultivar	Taiwan	TWN
61	IT221787	landrace	Japan	JPN
62	IT221789	landrace	Japan	JPN
63	IT227012	landrace	South Korea	KOR
64	IT227013	landrace	South Korea	KOR
65	IT227906	landrace	Uzbekistan	UZB
66	IT228181	landrace	Japan	JPN
67	IT228195	landrace	Peru	PER
68	IT228854	landrace	Uzbekistan	UZB
69	IT235343	landrace	South Korea	KOR
70	IT235413	landrace	Germany	DEU
71	IT235966	landrace	Kyrgyzstan	KGZ
72	IT247931	landrace	China	CHN
73	IT247932	Cultivar	China	CHN
74	IT247938	Cultivar	China	CHN
75	IT247960	landrace	China	CHN
76	IT247966	Cultivar	China	CHN
77	IT247979	Cultivar	China	CHN
78	IT248400	Cultivar	Japan	JPN
79	IT248599	landrace	South Korea	KOR
80	IT251123	Cultivar	China	CHN
81	IT260815	Cultivar	China	CHN
82	IT278620	landrace	Myanmar	MMR
83	IT278640	landrace	South Korea	KOR

84	IT278703	landrace	South Korea	KOR
85	IT293003	landrace	Mongolia	MNG
86	IT293004	landrace	Mongolia	MNG
87	IT293076	landrace	Austria	AUT
88	IT321040	landrace	South Korea	KOR
89	IT321041	landrace	South Korea	KOR
90	IT327479	Cultivar	China	CHN
91	IT336315	landrace	South Korea	KOR
92	IT339601	Cultivar	South Korea	KOR
93	IT339602	landrace	South Korea	KOR
94	IT344732	landrace	Malaysia	MYS
95	IT344733	landrace	Japan	JPN
96	IT344734	landrace	Japan	JPN
97	IT344735	landrace	Newzealand	NZL
98	IT344736	landrace	Austria	AUT
99	IT344737	landrace	Austria	AUT
100	IT344740	landrace	France	FRA
101	IT344741	landrace	Netherland	NLD
102	IT344742	landrace	United Kingdom	GBR
103	IT344743	Cultivar	Russia	RUS
104	IT344746	Cultivar	China	CHN
105	IT344747	Cultivar	China	CHN
106	IT344748	Cultivar	China	CHN
107	IT344749	Cultivar	China	CHN
108	IT344750	Cultivar	China	CHN
109	IT344751	Cultivar	Russia	RUS
110	IT344752	landrace	China	CHN
111	IT344755	landrace	Japan	JPN
112	IT344756	landrace	China	CHN
113	IT344757	Cultivar	South Korea	KOR
114	IT344758	Cultivar	South Korea	KOR
115	IT344759	Cultivar	South Korea	KOR

116	IT344760	Cultivar		South Korea	KOR
117	IT344761	Cultivar		South Korea	KOR
118	IT344763	landrace		China	CHN
119	IT344764	Cultivar		South Korea	KOR
120	IT344765	Cultivar		South Korea	KOR
121	IT344766	Cultivar		South Korea	KOR
122	IT344767	Cultivar		South Korea	KOR
123	IT344768	Cultivar		South Korea	KOR
124	IT344770	Cultivar		South Korea	KOR
125	IT344771	landrace		China	CHN
126	IT344772	landrace		China	CHN
127	IT344773	landrace		China	CHN
128	IT344774	landrace		China	CHN
129	IT344775	landrace		Thailand	THA
130	IT344776	landrace		Thailand	THA
131	IT344777	landrace		Thailand	THA
132	IT344778	landrace		South Korea	KOR
133	IT344779	landrace		South Korea	KOR
134	IT344780	Landrace		South Korea	KOR

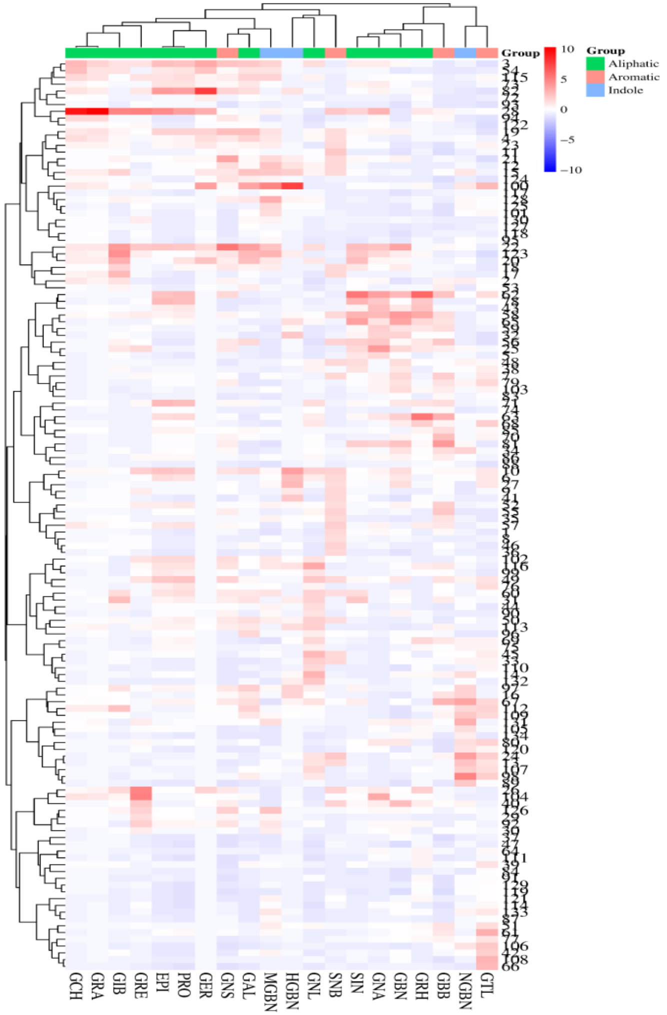

Figure S1. Heatmap hierarchical clustering showing glucosinolate compounds in 150 Chinese cabbage accessions. Color scale for hierarchical clustering is labeled. PRO: Progoitrin; EPI: Epiprogoitrin; GNL: Gluconapoleiferin; MGBS: 4-Methoxyglucobrassicin; GNS: Gluconasturtiin; GAL: Glucoalyssin; GRA: Glucoraphanin; GER: Glucoerucin; GBN: Glucobrassicanapin; GNA: Gluconapin; GCH: Glucocheirolin; SIN: Sinigrin; GTL: Glucotropaeolin; GRE: Glucoraphenin; GRH: Glucoraphasatin; GIB: Glucoiberin; SNB: Sinalbin; NGBS: Neoglucobrassicin; GBB: Glucobarbarin; HGBS: 4-Hydroxyglucobrassicin; TGSL: Total GSL
